# Establishment of a Mouse Ovarian Cancer and Peritoneal Metastasis Model to Study Intraperitoneal Chemotherapy

**DOI:** 10.3390/cancers12123818

**Published:** 2020-12-17

**Authors:** Günther A. Rezniczek, Jonathan Buggisch, Julien Sobilo, Alexandre Launay, Stéphanie Lerondel, Alain Le Pape, Mehdi Ouaissi, Daniel Göhler, Metin Senkal, Urs Giger-Pabst, Clemens B. Tempfer

**Affiliations:** 1 Department of Obstetrics and Gynecology, Marien Hospital Herne, Ruhr-Universität Bochum, 44625 Herne, Germany; clemens.tempfer@rub.de; 2 Department of General-, Visceral- and Transplant Surgery, Universität Münster, 48149 Münster, Germany; j_bugg01@uni-muenster.de (J.B.); ursgiger@gmx.net (U.G.-P.); 3 CNRS UPS44, CIPA, PHENOMIN-TAAM, 45071 Orléans, France; julien.sobilo@cnrs-orleans.fr (J.S.); alexandre.launay@cnrs-orleans.fr (A.L.); stephanie.lerondel@cnrs-orleans.fr (S.L.); alain.lepape@univ-tours.fr (A.L.P.); 4 Department of Digestive, Oncological, Endocrine, Hepato-Biliary, Pancreatic and Liver Transplant Surgery, University Hospital of Tours, 37044 Tours, France; mehdi.ouaissi@univ-tours.fr; 5 Research Group Mechanical Process Engineering, Institute of Process Engineering and Environmental Technology, Technische Universität Dresden, 01069 Dresden, Germany; daniel.goehler@gmx.de; 6 Topas GmbH, 01237 Dresden, Germany; 7 Department of Surgery, Marien Hospital Witten, 58452 Witten, Germany; metin.senkal@elisabethgruppe.de

**Keywords:** pressurized, intraperitoneal, aerosol, chemotherapy, peritoneal, metastasis, ovarian cancer, mouse model

## Abstract

**Simple Summary:**

Intraperitoneal chemotherapy (IPC) is a locoregional treatment option in patients with peritoneal metastases (PM). Small animal models are valuable research tools allowing for rapid, reproducible, and inexpensive study and optimization of different forms of IPC. Here, we present a mouse model of ovarian cancer-derived PM and demonstrate its suitability for various modes of IPC, including pressurized intraperitoneal chemotherapy (PIPAC) using a micro-nozzle for aerosolized drug delivery.

**Abstract:**

Intraperitoneal chemotherapy (IPC) is a locoregional treatment option in patients with peritoneal metastases (PM). Here, we present an ovarian cancer (OC)-derived PM mouse model for the study of different forms of IPC. Xenograft cell proliferation (luciferase-transfected OVCAR3 and SKOV3 clones) and growth kinetics were assessed using PET scan, bioluminescence imaging (BLI), and histological tumor analysis. Liquid IPC was achieved by intraperitoneal injection with/without capnoperitoneum (6–7 mmHg). Pressurized intraperitoneal aerosol chemotherapy (PIPAC) was mimicked using an intratracheal drug aerosol administration system (micro-nozzle), which, as demonstrated by ex vivo granulometric analysis using laser diffraction spectrometry, produced a polydisperse, bimodal aerosol with a volume-weighted median diameter of (26.49 ± 2.76) µm. Distribution of Tc-99m-labeled doxorubicin in mice was characterized using SPECT and was dependent on the delivery mode and most homogeneous when the micro-nozzle was used. A total of 2 mg doxorubicin per kg body weight was determined to be the optimally effective and tolerable dose to achieve at least 50% tumor reduction. Repeated PIPAC (four times at seven-day-intervals) with doxorubicin in SKOV3-luc tumor-bearing mice resulted in halted tumor proliferation and tumor load reduced after the second round of PIPAC versus controls and the number of tumor nodules was significantly reduced (27.7 ± 9.5 vs. 57.3 ± 9.5; *p* = 0.0003). Thus, we established the first mouse model of OC PM for the study of IPC using a human xenograft with SKOV3 cells and an experimental IPC setup with a miniaturized nozzle. Repeated IPC was feasible and demonstrated time-dependent anti-tumor activity.

## 1. Introduction

Ovarian cancer (OC) is a deadly disease with high morbidity and mortality affecting between 3 and 11 per 100,000 women worldwide [1]. OC is typically diagnosed at a late stage with peritoneal metastases (PM) being diagnosed in around 75% of cases [2]. The high proportion of women with PM at initial diagnosis results in a poor prognosis with 5-year and 10-year survival rates of only 40% and 20%, respectively [3]. PM is the key element in the locoregional growth and progression of OC resulting in a cascade of symptoms including ascites, malnutrition, bowel obstruction, cachexia, and ultimately death [4,5]. Therefore, many aspects of OC treatment focus on the peritoneal cavity in order to clear the peritoneum from tumor cells and to prevent PM growth and re-growth. Specifically, surgery, systemic chemotherapy, and intraperitoneal chemotherapy (IPC) are the available means of treating PM in women with OC [2,6,7]. IPC strategies include intravenous/intraperitoneal combination chemotherapy, hyperthermic intraperitoneal chemotherapy (HIPEC), and pressurized intraperitoneal aerosol chemotherapy (PIPAC). Intravenous/intraperitoneal chemotherapy was introduced in 1996 and is a combination of traditional intravenous chemotherapy and repeated intraperitoneal instillations of cisplatin or carboplatin and paclitaxel as an adjuvant therapy after upfront cytoreductive surgery [7,8]. HIPEC, introduced in 1978, is a heated liquid IPC, which is used as a one-stop strategy together with cytoreductive surgery either in the neo-adjuvant, adjuvant, or recurrent setting [9]. Chemotherapy compounds most often used for HIPEC in OC patients are cisplatin, doxorubicin, and mitomycin [9,10]. Lastly, PIPAC is the most recent form of IPC using repeated laparoscopies to apply aerosolized and pressurized chemotherapy compounds such as cisplatin and doxorubicin in patients with PM from OC and various other origins [11,12]. All three forms of IPC have been extensively studied in clinical trials, but controversy remains as to their efficacy, optimal use, and sequence. One of the most important limitations of IPC is its local toxicity. Chemical peritonitis, pain, gastrointestinal side effects, and catheter-associated complications have limited the widespread use of IPC [7,8,9,10]. More research is needed to define the optimal conditions of IPC in order to benefit from its high locoregional efficacy while reducing unwanted gastrointestinal and systemic toxicity. Thus, animal models allowing for rapid, reproducible, and inexpensive study of different forms of IPC are warranted. Recently, van de Sande et al. developed an animal model of IPC using Wistar rats undergoing laparoscopy with capnoperitoneum, aerosolized chemotherapy, and electroprecipitation [13]. This tumor model used a xenograft of ovarian PM based on SKOV-3 luciferase-positive cells. Miliary PM was established and a single application of a laparoscopic IPC procedure was feasible. In a follow-up study using the same animal model, Shariati et al. demonstrated that nanoparticles carrying chemotherapeutics such as cisplatin-loaded polyarginine-hyaluronic acid nanoscale particles can be effectively applied to the peritoneum via IPC [14]. However, although these works demonstrate that a rodent model of IPC is feasible, the tolerability of repeated IPC courses has not been established in the rat model. A mouse model of OC PM allowing for a quicker and cheaper study of IPC compared to a rat model is not available.

Therefore, we undertook to establish a mouse model of OC PM with luciferase-transfected clones of ovarian cancer cell lines in nude mice and an IPC setup for the application of liquid IPC, capnoperitoneum-liquid IPC, and PIPAC using a miniaturized nozzle for drug delivery. The aim of our study was to demonstrate that repeated IPC using various forms of drug delivery is feasible in mice within the context of an OC PM model.

## 2. Results

### 2.1. Mouse Ovarian PM Model

**Figure 1:**
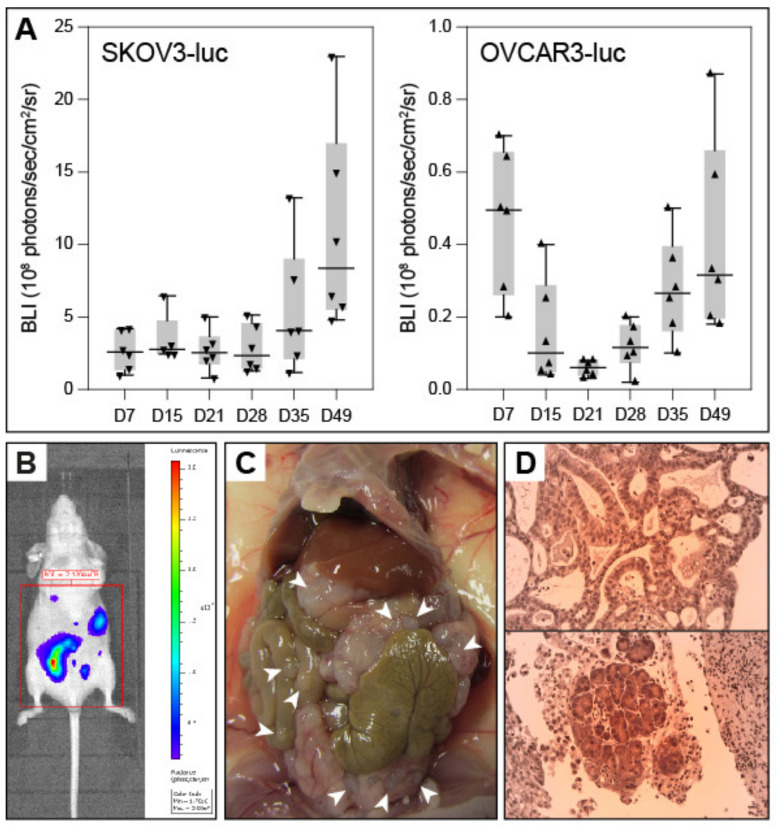
Mouse ovarian peritoneal metastasis model. (**A**) Tumor growth (SKOV3-luc, left panel; OVCAR3-luc, right panel; *n* = 6 mice per cell line) measured by bioluminescence imaging (BLI) over 7 weeks. Box plots: horizontal line, median; box, 25/75-th percentile; whiskers, 10/90-th percentile; triangles, individual data points. (**B**) Representative BLI quantification (SKOV3-luc). (**C**) Representative necroscopy showing numerous tumor nodules (arrowheads). (**D**) Representative histopathology images (HES staining) showing massive peritoneal infiltration by a solid and glandular tumor mass containing some focal necrosis (upper panel). Focally, a micropapillary pattern is also observed (lower panel). Tumor cells display a severe anisokaryosis and numerous abnormal mitotic figures, and in some areas, clear cells are observed (both panels). Magnification: ×200.

Two cell lines (SKOV3 and OVCAR3) were evaluated for producing an OC-PM mouse model. Stable luciferase-expressing cell lines have been established and both cell lines led to tumor growth after intraperitoneal injection into BALB/c nude mice (*n* = 6 for each cell line). Growth characteristics, both in culture and after transplantation (measured at several time points by BLI), favored SKOV3-luc over OVCAR3-luc (Figure 1A,B). Upon autopsy on day 61 after transplantation, several larger (>1 mm^3^) and many small tumors were present in SKOV3-luc nude mice (Figure 1C), whereas only a few discrete tumors were found in OVCAR3-luc nude mice (not shown). HES staining confirmed the presence of tumor cells in the nodules (Figure 1D), with characteristics similar to those observed in peritoneal metastases of ovarian high-grade serous carcinoma in humans, i.e., significant nuclear atypia and pleomorphism, a solid growth pattern, a scattered invasion front, and necrosis. Thus, SKOV3-luc was chosen for further experiments.

**Figure 2:**
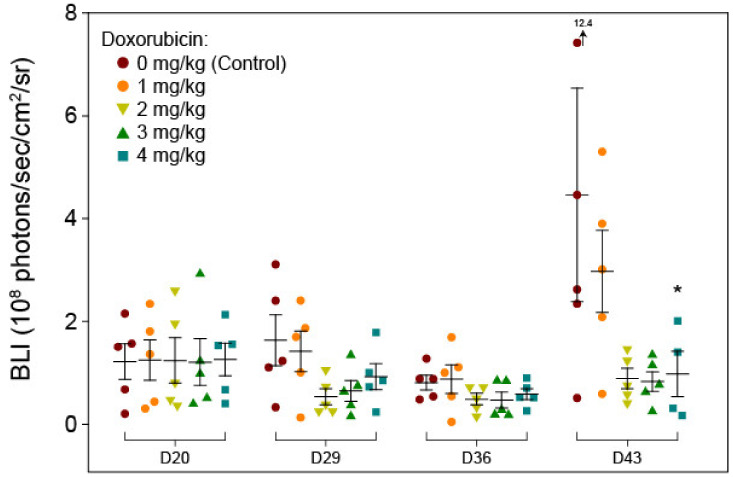
ID50 determination via quantification of bioluminescence imaging data (measured on the days 20, 29, 36, and 43 after tumor cell injection). Symbols represent data from individual mice in the various treatment groups (*n* = 5 per group). Doxorubicin was injected intraperitoneally without a capnoperitoneum on days 21, 28, 25, and 42. Horizontal bars and whiskers represent the means and standard errors of the mean, respectively. *, in the 4 mg/kg body weight group, one mouse died after the last injection (before imaging) and a second mouse in this group died before autopsy. D, day.

To identify the target dose for PIPAC, we first determined the optimal target dose that resulted in an about 50% inhibition (ID_50_) of metastasis proliferation versus an untreated control group in the SKOV3-luc PM mouse model. To this end, we intraperitoneally injected mice bearing SKOV3-luc-derived tumors with different concentrations of doxorubicin (0 = control, 1, 2, 3, and 4 mg/kg body weight; *n* = 5 mice per group) once per week over four weeks, starting on day 21 after tumor cell injection. Quantification of BLI data over the course of the experiment is shown in Figure 2. In the 4 mg/kg-body-weight group, 2/5 mice died after the last doxorubicin injection. Thus, based on these results, 2 mg doxorubicin per kg body weight was determined to be the optimally effective and tolerable dose to achieve at least 50% reduction in tumor proliferation.

### 2.2. Micro-Nozzle for Aerosolized Delivery into a Capnoperitoneum

**Figure 3:**
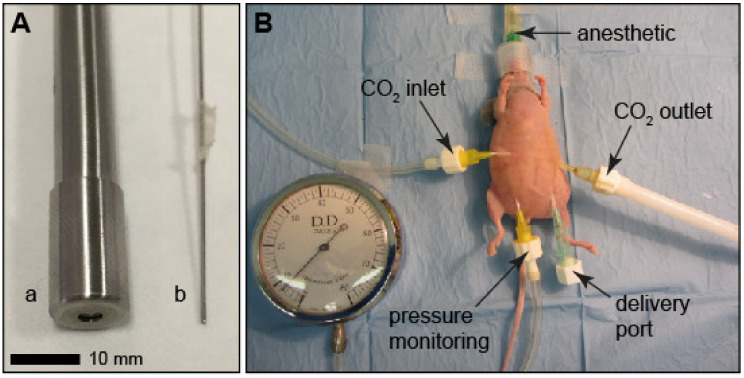
Setup for pressurized intraperitoneal aerosol chemotherapy (PIPAC) delivery in mice using the micro-nozzle. (**A**) Comparison of the CapnoPen (**a**) and the micro-nozzle (**b**). Scale bar, 10 mm. (**B**) PIPAC setup showing an anesthetized BALB/c nude mouse placed on a heating pad (under the blue cover) and with all ports (CO_2_ inlet and outlet, pressure monitoring, and the delivery port through which the micro-nozzle is inserted).

In order to simulate PIPAC in this mouse model, due to the large geometric dimensions of the standard PIPAC nozzle (i.e., an outer diameter of approx. 12 mm), a manually operated commercial intratracheal drug aerosol administration system (MicroSprayer^®^ Aerosolizer nozzle Model 1A–1C and high pressure syringe FMJ-250, Penn-Century, Philadelphia, PA, USA) for small animals (outer diameter approx. 0.7 mm with 10 µm aperture) [15] was used. Figure 3A shows the micro-nozzle in comparison to the standard injection device for human use (CapnoPen^®^), and Figure 3B shows the experimental setup for mice.

**Figure 4:**
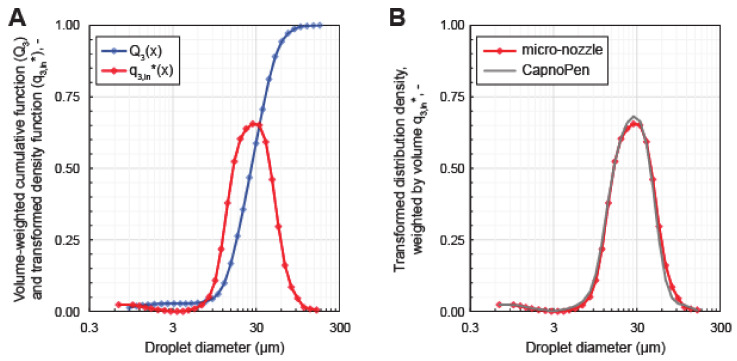
Granulometric analysis. (**A**) Volume-weighted droplet size distribution of the micro-nozzle (means, *n* = 3) as measured by laser diffraction spectrometry. (**B**) Comparison of the volume-weighted droplet size distributions of the micro-nozzle (based on aerosolization of a 0.9 wt.-% aqueous sodium chloride solution (present study) to that of the standard PIPAC nozzle for the use in humans (CapnoPen^®^, operated with a 5.0 wt.-% aqueous glucose solution as reported in [16]). Graphical representation according to ISO 9276-1:1998 [17].

To characterize the drug aerosol produced by the micro-nozzle, ex vivo granulometric aerosol analysis was performed by means of laser diffraction spectrometry. Figure 4A shows the mean (*n* = 3) volume-weighted droplet size distribution at the outlet of the micro-nozzle. Thus, the micro-nozzle produced a polydisperse, bimodal aerosol with a volume-weighted median diameter of x_50,3_ = (26.49 ± 2.76) µm (corresponding to the mass median diameter for the nebulized liquid with quasi unit density of approx. 1 g/mL). The size distribution showed a local minimum at 3 µm with a volume-weighted fine fraction of Q_3_ (3 µm) = 2.75 vol.-% and a volume-weighted coarse fraction of 1-Q_3_ (3 µm) = 97.25 vol.-%. Thus, the spray characteristics of this micro-nozzle were equivalent to those of the CapnoPen^®^ (Figure 4B).

### 2.3. Intraperitoneal Distribution of Doxorubicin

**Figure 5:**
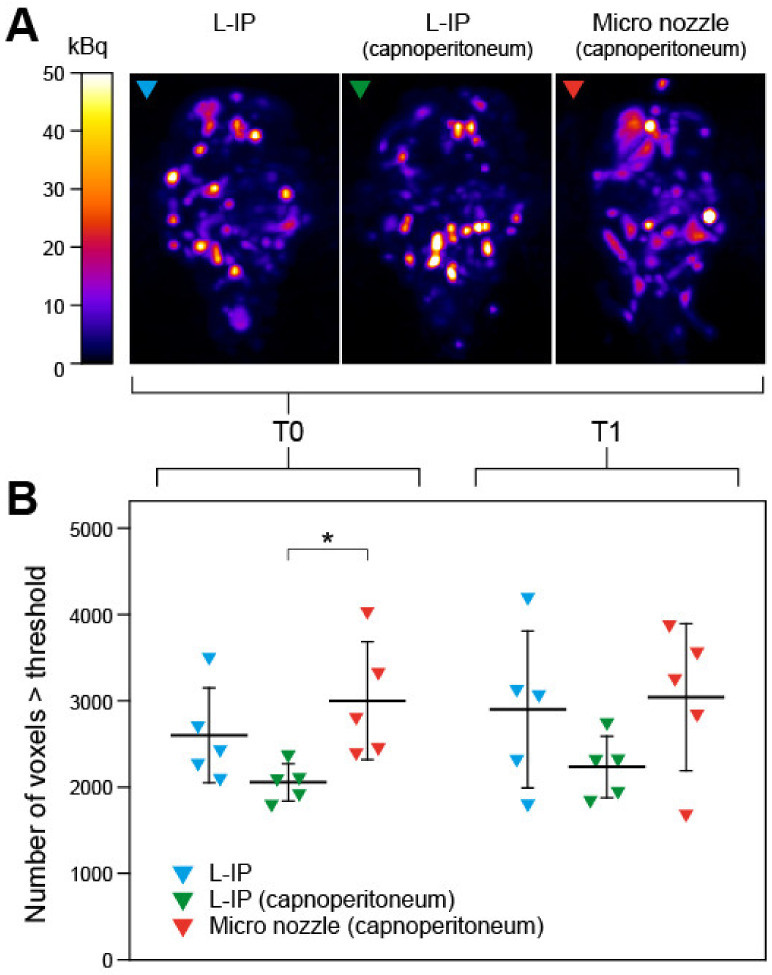
SPECT analysis of the intraabdominal distribution pattern of Tc-99 m-labeled doxorubicin. (**A**) Representative SPECT scans of mice in anterior-posterior maximum intensity projection (MIP) at T0. The NIH Image Fire2 pallete was used and range was set to 0–50 kBq. (**B**) Distribution, represented by number of voxels exceeding a threshold activity of 5 kBq (T0) or 4 kBq (T1; see Materials and Methods). Higher values correspond to a more uniform distribution pattern. Triangles represent individual mice (*n* = 5), thick horizontal lines are means, error bars represent standard deviations. L-IP, liquid intraperitoneal injection; T0, 20 min after administration of doxorubicin; T1, T0 + 1 h. Statistical significance is indicated: *, *p* < 0.05.

Next, we used SPECT imaging to assess the spatial distribution of radiolabeled (Tc-99 m) doxorubicin delivered to the capnoperitoneal cavity via the micro-nozzle (aerosol/spray, *n* = 5) versus simple injection as a liquid (L-IP), with or without capnoperitoneum (*n* = 5 each) (Figure 5A). A stable capnoperitoneum with a capnoperitoneal pressure of 6–7 mmHg could be established within 10 to 15 s after initiation of a constant CO_2_ flow rate of 60 mL/min. No manual readjustment of the CO_2_ flow was necessary. Prolonged capnoperitoneum (up to 30 min) was well tolerated by all animals. For longer durations (up to 1 h) it was observed that using pure oxygen instead of air for gaseous anesthesia was required to avoid any signs of cardio-respiratory distress. Quantitative analysis of the SPECT data revealed that 20 min after the administration of the radiolabeled compound (T0), distribution (as measured by the number of voxels exceeding a threshold activity; see Materials and Methods for details) was most homogeneous in the case of delivery through the micro-nozzle into a capnoperitoneum (3001 ± 685 vs. 2056 ± 217 in case of L-IP into a capnoperitoneum, *p* = 0.042; and 2601 ± 550 in case of L-IP without capnoperitoneum, not significant, respectively). At T1 (T0 +1 h), the homogeneity of the distribution pattern slightly increased in all groups and there were no significant differences among the delivery modes (Figure 5B).

### 2.4. PIPAC Treatment

**Figure 6:**
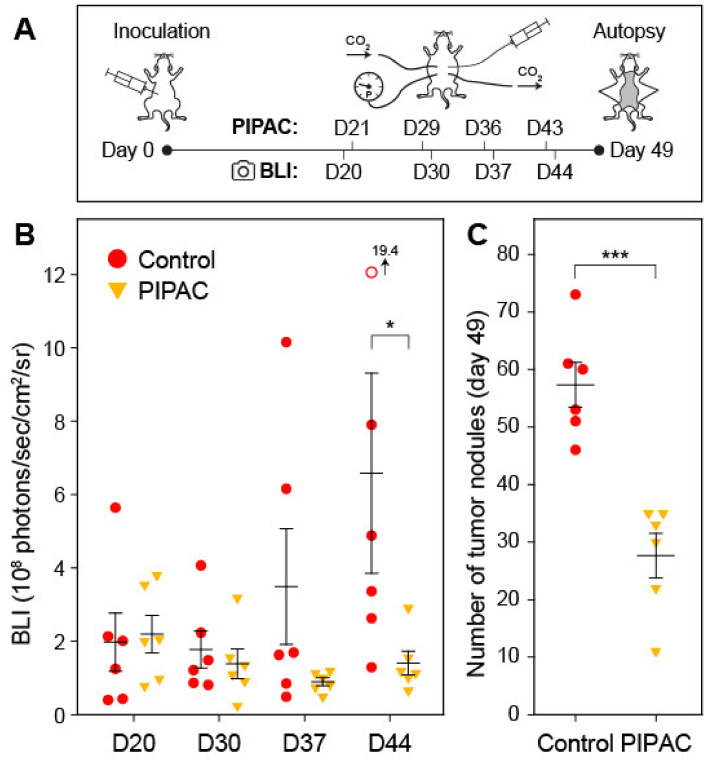
Repeated PIPAC in an ovarian peritoneal metastasis mouse model. (**A**) Experimental timeline. Mice (*n* = 6 per group) were stratified into treatment and control groups based on bioluminescence imaging (BLI) results on day 20 (D20). (**B**) Quantification of BLI data from control (red circles; the open circle represents a value outside the graphs scale) and PIPAC-treated mice (orange triangles). (**C**) Macroscopic necroscopy results showing the number of tumor nodules identified in the control mice (red circles) and PIPAC-treated mice (orange triangles) on day 49. Horizontal bars and whiskers represent the means and standard errors of the mean, respectively. Statistical significance (one-tailed t-test) is indicated: *, *p* < 0.05; ***, *p* < 0.001.

To demonstrate the feasibility of this mouse model for studying anti-tumor efficacy of PIPAC, we subjected SKOV3-luc tumor-bearing mice to a series of four treatments at 7-day-intervals (starting 21 days after injection of tumor cells), with either doxorubicin (2 mg/kg body weight in 200 µL PBS; treatment group, *n* = 6) or excipient alone (200 µL PBS; control group, *n* = 6) delivered via the micro-nozzle into a capnoperitoneum. Figure 6A gives an overview of the timeline of the experiment. Tumor load was quantified (BLI) before the first and after each further treatment. As shown in Figure 6B, tumor proliferation was halted in the treatment group and tumor load reduced after the second round of PIPAC, while considerable proliferation and increased tumor load was found in the control group. Necroscopy revealed that the number of tumor nodules was significantly reduced in the treatment group (27.7 ± 9.5 vs. 57.3 ± 9.5; t-test: *p* = 0.0003; Figure 6C).

## 3. Discussion

OC is a common and deadly malignant disease, which typically spreads to the peritoneal cavity [1,2]. IPC is an established means of OC treatment besides surgery and systemic chemotherapy [1,2,3,4,5,6]. There is, however, no mouse model available to allow for the experimental study of IPC within the context of OC with PM (literature search using the PubMed database with search terms: intraperitoneal chemotherapy, HIPEC, PIPAC, mouse model, animal model; search date: 20 October 2020). Here, we describe the development of a feasible, reproducible, and reliable mouse model using a human xenograft OC cell line and an experimental IPC setup mimicking human liquid IPC and IPC based on capnoperitoneum such as PIPAC. We were able to (1) demonstrate that luciferase-expressing SKOV3 in BALB/c nude mice showed constant and reproducible intraperitoneal tumor growth, (2) establish the optimally effective and tolerable dose to achieve a 50% tumor reduction (2 mg doxorubicin per kg body weight), (3) establish the superiority of PIPAC regarding the spatial distribution of a radiolabeled compound in the mouse abdomen compared to liquid-IPC with or without capnoperitoneum, and finally (4) show that four rounds of consecutive PIPACs with doxorubicin significantly reduced OC PM tumor load. The results of our study establish the first mouse model for IPC within the context of OC with PM. Using this animal model, we can now subsequently compare different chemotherapy compounds, concentrations, and combinations under various IPC conditions modeling liquid IPC, HIPEC, and PIPAC.

Establishing a stable mouse model of IPC is difficult, especially when a capnoperitoneum is needed in order to mimic laparoscopically delivered IPC in humans. In contrast to large animals such as pigs and sheep, mice are far less tolerable of a capnoperitoneum and there is no standard technique available for building up the capnoperitoneum in a mouse and there are no adequate established access devices such as trocars and nozzles for mice [18]. Therefore, in our model, we had to miniaturize the standard PIPAC nozzle and thus, selected a commercial micro-nozzle originally designed for intratracheal drug aerosol administration [19]. We then successfully demonstrated that this device produced a polydisperse, bimodal aerosol with a volume-weighted median diameter of 26 µm closely resembling the granulometric characteristics of the spraying device normally used in humans. In addition, we were able to show that our IPC mouse model produced constant distribution patterns within the peritoneal cavity with clear differences depending on the type of IPC used. In addition, we identified the optimum time frame within which distribution of intraperitoneal compounds reached their optimum. Of note, we found that PIPAC had the best distribution pattern among the three different forms of IPC studies, which is consistent with data found in the rat model [13] and in humans [20]. This suggests that our mouse model is an adequate surrogate for studying human IPC. Another interesting finding was that in some mice, a pronounced spot of higher compound concentration in areas opposite the tip of the micro-nozzle (‘impaction point’) was identified. This finding is also consistent with previously published ex-vivo studies identifying varying depths of chemotherapy infiltration depending on the trajectory of the IPC application device [21]. The clinical impact of this finding regarding anti-tumor efficacy and local complications is unclear and should be the focus of future studies.

Our study has limitations. First, the morphological aspect of intraperitoneal carcinomatosis as established in our model is bulky as opposed to the miliary spread seen in most cases of human PM. Thus, tumor efficacy studies might not be representative of miliary PM. Second, we did not observe the production of ascites in our mice. This might be of importance since >80% of human patients with PM present with ascites and ascites is known to be a transporter and promoter of intraabdominal PM spread [1,2]. Future studies should address this issue appropriately, for example, by abdominal flushings or tumor fluid instillations. Third, the ovarian cancer cell line used in our experiments was SKOV-3. Based on gene expression studies, SKOV-3 may not be the best cell line regarding its genetic similarity to high-grade serous cancers [22]. Therefore, alternative ovarian cell lines should be explored in the future when expanding our IPC mouse model.

Our tumor mouse model of OC PM was stable and we demonstrated time-dependent PM tumor growth. In addition, we established the optimally effective and tolerable dose to achieve a 50% PM tumor reduction. This suggests that the mouse model presented in this study is successfully representing PM from OC and can measure the anti-tumor effects of IPC. Specifically, we were able—for the first time—to show that repeated IPC in the setting of a capnoperitoneum using a miniaturized pressure delivery nozzle is tolerated by mice. Therefore, our mouse model can be used to study PIPAC as well as HIPEC and liquid IPC. Future experiments may examine the anti-tumor efficacy of different chemotherapy compounds and targeted therapies such as antibodies and their combinations in the context of IPC. In addition, this mouse model should be sufficient to study the fine-tuning and optimization of IPC conditions regarding chemotherapy concentrations, pressure conditions, and capnoperitoneal variations. These experiments may be able to improve our knowledge of how to make the best use of IPC in humans.

## 4. Materials and Methods

### 4.1. Animals

All mouse experiments were carried out at the CIPA, PHENOMIN-TAAM facility (CNRS Orléans, France). BALB/c nude mice (Charles River, France; strain code 194; CAnN.Cg-*Foxn1^nu^*/Crl) were used for induction of PM, and BALB/cByJ (Charles River; strain code 627) were used to study biodistribution. Mice were kept in standard conditions (water/food ad libitum, 12-h light/dark cycle) and handled and cared for in accordance with national and institutional guidelines. All experiments have been approved by the Comité d’Ethique de la Région Centre Val de Loire n°3 and by The Ministry of Higher Education, Research and Innovation, France (Apafis N°23624).

### 4.2. Ovarian Cancer Peritoneal Metastasis Mouse Model

Stable firefly luciferase (luc)-transfected clones of the ovarian cancer cell lines OVCAR3 (ATCC HTB-161; HER2-negative; medium: RPMI-1640 supplemented with 0.01 mg/mL insulin and 20% FBS) and SKOV3 (ATCC HTB-77; HER2-positive; medium: McCoy’s 5a supplemented with 10% FBS) were generated by transfection with a vector containing the luc gene and a selection cassette (ready-to-use lentivirus particles expressing luciferase under the control of the EF1a promoter with blasticidin selection marker; catalog no. LVP433-PBS, amsbio, UK) using standard techniques. The EF1a promotor was found most suitable (over CMV, PKG, and UbC promotors) in preliminary experiments using the lentiviral promotor Blast kit (catalog no. LV950, Applied Biological Materials Inc., Canada) [23]. After selection/expansion, clones were tested for the stability of luc expression and bioluminescence intensity per cell. Imaging of 1 × 10^5^ cells in 200 µL culture medium with 30 µg D-luciferin was performed in an IVIS Lumina III imaging system (PerkinElmer, France). Suitable clones (SKOV3-luc: 6.4 × 10^6^ photons/sec/cm^2^/sr; OVCAR3-luc: 3.0 × 10^6^ photons/sec/cm^2^/sr) were further expanded and stored as aliquots in liquid nitrogen.

To establish tumor engraftments, female BALB/c nude mice were injected with 1 × 10^7^ cells (in 200 µL) intraperitoneally at two contra lateral sites (100 µL with 5 × 10^6^ cells each) and proliferation and growth kinetics of the engrafted metastases were monitored using positron emission tomography (PET) and/or bioluminescence imaging (BLI). Four weeks post induction of PM, mice were sacrificed by overdose of anesthesia and autopsied to determine the number of metastases and to compare their anatomical locations to foci observed by BLI and PET scans.

### 4.3. Positron Emission Tomography (PET) and Bioluminescence Imaging (BLI)

For PET assessment, ^18^FDG (AAA, France; activity of approx. 15 MBq) was injected into the tail veins of mice, which had free access to water but not food for 12 h before the examination. After 1 h of tracer distribution in the body, animals were placed in the bed of a micro PET scanner (eXplore VISTA, Sedecal, Spain). To get whole body images, scans were acquired over 20 min using an energy window setting of 250–700 keV. Radionuclide decay was automatically corrected between each bed position. Images were reconstructed and visualized using MMWKS Image Software (4.7 Build 452), using the 3D FORE/2D OSEM algorithms.

Bioluminescence imaging (BLI) was performed using the IVIS Lumina III instrument (PerkinElmer, France). Mice received an injection of luciferin (2 mg in 100 µL) 4 min before induction of anesthesia (2% air/isoflurane mixture). Animals were imaged at both posterior and anterior faces and quantification of bioluminescence was performed with the Living Image 4.4 software (PerkinElmer). Considering the known limitation of BLI about the resolution of deep foci inside the abdomen that makes it impossible to quantitate the evolution of each metastasis, in a preliminary study, we had performed complete assessment of metastatic activity by ^18^FDG-PET and found BLI to be adequate for routine quantification of tumor load in nude mice [23].

### 4.4. Necroscopy and Histology

Mice were sacrificed by cervical dislocation immediately after the last imaging procedure while still under anesthesia and autopsied to determine the number of metastases and to compare their anatomical locations to foci observed by BLI. Tumor nodules were fixed in 10% formalin solution, dehydrated in ethanol, and embedded in paraffin for standard hematoxylin-eosin-saffron (HES) staining.

### 4.5. Granulometric Analysis

A laser diffraction spectrometer (HELOS/KR-H2487, Sympatec GmbH, Germany) according to ISO 13320:2009 [24] was operated for determining volume-weighted droplet size distributions over a size range from 0.5–175 µm [25]. Analogous to previous studies [16,26], aerosol characterization was performed at a distance of approx. 5 mm from the aerosol outlet of the nebulizer. For the purpose of analysis, the aerosol generation system was operated with a 0.9 wt.-% aqueous sodium chloride solution (B. Braun Melsungen AG, Melsungen, Germany). Measurements were performed three times over the whole aerosolization time of approx. 5 s.

### 4.6. Characterization of Spatial Drug Distribution in Mice

To assess compound distribution in the peritoneal cavity of mice (BALB/c ByJ), we used Tc-99m-labeled doxorubicin, which was produced as follows: 0.9 mg doxorubicin·HCl (Teva, Germany) was pre-mixed with 120 µg SnF_2_ (Sigma Aldrich, St. Quentin Fallavier, France) in a total volume of 1 mL oxygen-free saline in a vacuum flask (“pre-tinning process”) for 5 min before adding 1.8 GBq [^99m^Tc] sodium pertechnetate in saline and adjusting the final volume to 3.6 mL. This resulted in a >96% radio chemical purity of doxorubicin as assessed by thin layer chromatography [23]. Portions of 90–110 MBq (adjusted over time to compensate for decay, approx. 200 µL and corresponding to within 2–3 mg/kg body weight doxorubicin in mice of approx. 25–28 g weight) were used per mouse and either directly injected into the peritoneal cavity using a syringe (IP injection) or delivered via the micro-nozzle as described above. In both groups, a capnoperitoneum with a capnoperitoneal pressure of 8-10 cmH_2_O (5.8–7.3 mmHg) was maintained during the application and for 20 min afterward. Then, SPECT (Single Photon Emission Computed Tomography) was performed (without capnoperitoneum). Mice were kept under 2.0% isoflurane (Iso-Vet, Piramal Healthcare, Morpeth, UK) anesthesia in air carrier at 0.5 L/min and maintained at 37 °C during the whole procedure (injection, incubation, imaging). A dedicated 4-heads, multiplexed multi-pinhole small animal SPECT/CT imaging device (NanoSPECT/CT; Mediso, Budapest, Hungary) was used. The system was equipped with 4 collimators (nine pinholes, aperture 1.5 mm). The detection energy window for Tc-99 m was set at 140 keV ± 10%. Helical scanning mode was used and 24 projections of 30 s were acquired resulting in 9-min scans. The imaged data (91 × 86 × 86 voxels) were reconstructed with HiSPECT NG software (Scivis GmbH, Göttingen, Germany), analyzed with InVivo Scope image analysis software (Bioscan Inc., USA), and stored in Digital Imaging and Communications in Medicine (DICOM) files. For quantitative spatial distribution analysis, histogram data (1000 bins, ranging from 0–100 kBq) were exported from VivoQuant 4.0 (Invicro, Boston, MA) and the numbers of voxels exceeding a threshold activity were determined (5 kBq at T0 and 4 kBq at T1, respectively, in order to correct for the decay; total measured activity in *n* = 15 mice at T1 was 80.7% ± 9.5%). Higher numbers correspond to a more uniform distribution. SigmaPlot14 (Systat Software Inc., San Jose, CA, USA) was used for statistical analysis. The Shapiro–Wilk test was chosen to assert normal distribution of experimental data, and the Brown–Forsythe test to assert equal variance. ANOVA was used to compare experimental groups. All reported *p*-values are two-tailed.

### 4.7. PIPAC in Mice

The experimental PIPAC procedure for mice was adapted from the clinical treatment protocol [27] and performed in a class II laminar flow hood. Mice were anesthetized (initial dose: 5% air/isoflurane mixture) and anesthesia was maintained (2%) throughout the whole procedure, during which mice were in a supine position (fixation of extremities was not necessary) on a heating pad set to 39 °C and body temperature was additionally maintained through a heating lamp. Four ports to the peritoneum were placed, i.e., (i) an inlet port for CO_2_, (ii) an outlet port for CO_2_, (iii) one port for monitoring the capnoperitoneal pressure, and iv) one port for drug delivery (Figure 3B). For the first three ports, standard cannulas (24G; Surflow I.V. 0.67 × 19 mm, Terumo Europe) were used, which were connected to the corresponding tubing by Luer lock adapters. CO_2_ flow was controlled by means of a variable area flow meter (model P; Aalborg, Orangeburg, NY, USA) and a capnoperitoneum with a capnoperitoneal pressure of 8–10 cmH_2_O (5.8–7.3 mmHg) was established. To deliver chemotherapeutics, the micro-nozzle was inserted through an 18G catheter (Surflow I.V. 1.30 × 32 mm, Terumo) that was closed with a Luer lock stopper while not in use. The desired chemotherapeutic dose was delivered administered in a total volume of 200 µL PBS. Care should be taken that the nozzle is inserted at a flat angle and parallel to the animal’s body axis in order to maximize the available distance/volume before the spray impacts on any tissue. The capnoperitoneum was maintained for 20 min after drug administration. At the end of the procedures, CO_2_ flow was stopped and cannulas were removed. Occasional bleeding was stopped with a drop of surgical glue. Within minutes of stopping anesthesia, mice were active and showed no indications of any adverse effects brought about by the procedure. Repeated PIPAC applications have been performed at 7-day-intervals for up to 4 times without any issues.

### 4.8. Data Handling and Statistics

Descriptive statistics are reported using means and standard deviations for normally distributed data and medians and interquartile ranges (IQR) for data not meeting this assumption. Accordingly, statistical analysis was performed using parametric (t-test) or nonparametric tests (Mann–Whitney U test). To compare rates and proportions, the χ^2^-test was used. All *P* values are two-tailed (unless noted otherwise) and *p* < 0.05 was considered statistically significant. We used the statistics software package SigmaPlot 14 (Systat Software Inc., San Jose, CA, USA) for statistical analysis.

## 5. Conclusions

In summary, we have established the first mouse model of OC with PM for the study of IPC using a human xenograft with SKOV3 as the OC cell line and an experimental IPC setup with a miniaturized nozzle as the application device. We were able to show linear PM growth, constant distribution patterns of injected IPC compounds within the peritoneal cavity with clear differences depending on the type of IPC used. Lastly, repeated IPC was feasible and demonstrated time-dependent anti-tumor activity.
